# Bis(2,2′-bi-1*H*-imidazole-κ^2^ *N* ^3^,*N* ^3′^)bis­(dimethyl sulfoxide-κ*O*)copper(II) bis­(tetra­fluoridoborate)

**DOI:** 10.1107/S1600536810031922

**Published:** 2010-08-18

**Authors:** Yong-Cheng Dai, Qiong-Hua Jin, Li-Na Cui, Li-Jun Xu, Cun-Lin Zhang

**Affiliations:** a Department of Chemistry, Capital Normal University, Beijing 100048, People’s Republic of China; b Beijing Key Laboratory for Terahertz Spectroscopy and Imaging, Key Laboratory of Terahertz Optoelectronics, Ministry of Education, Capital Normal University, Beijing 100048, People’s Republic of China

## Abstract

In the title copper(II) salt, [Cu(C_6_H_6_N_4_)_2_(C_2_H_6_OS)_2_](BF_4_)_2_, the Jahn–Teller distorted octa­hedral coordination sphere of copper is formed from four 2,2′-bi-1*H*-imidazole N atoms and two dimethyl sulfoxide O atoms. The Cu atom lies on a center of inversion. N—H⋯O and N—H⋯F hydrogen bonds give rise to a one-dimensional structure. The BF_4_^−^ anion is disordered over two sites in a 0.671 (10):0.329 (10) ratio.

## Related literature

Supra­molecular complexes containing H_2_biim (H_2_biim = 2,2′-biimidazole) have been applied widely in mol­ecular catalysis, photoelectric conversion materials and mol­ecular recognition, see: Ding *et al.* (2005 ▶). For the effect of the coordination bonds, inter­molecular hydrogen bonds and π–π packing inter­actions on the mol­ecular arrangement, see: Burrows (2004 ▶); Dai *et al.* (2009 ▶). For related structures, see: Jin *et al.* (2010 ▶); Aminou *et al.* (2004 ▶); Gruia *et al.* (2007 ▶); Yang *et al.* (2008 ▶). For Cu—O coordination bond lengths, see: Tao *et al.* (2002 ▶).
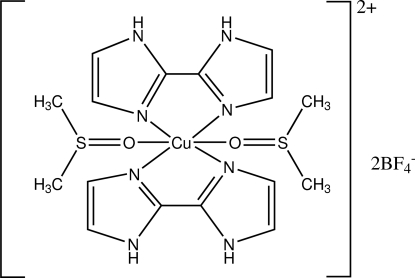


## Experimental

### 

#### Crystal data

[Cu(C_6_H_6_N_4_)_2_(C_2_H_6_OS)_2_](BF_4_)_2_
*M* *_r_* = 661.71
Triclinic, 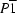

*a* = 7.059 (1) Å
*b* = 10.0721 (13) Å
*c* = 10.3669 (15) Å
α = 113.436 (2)°
β = 96.860 (1)°
γ = 92.000 (1)°
*V* = 668.68 (16) Å^3^
*Z* = 1
Mo *K*α radiation
μ = 1.06 mm^−1^
*T* = 298 K
0.36 × 0.32 × 0.20 mm

#### Data collection

Bruker SMART CCD area-detector diffractometer
Absorption correction: multi-scan (*SADABS*; Bruker, 2007 ▶) *T*_min_ = 0.701, *T*_max_ = 0.816
3418 measured reflections
2293 independent reflections
1885 reflections with *I* > 2σ(*I*)
*R*_int_ = 0.023

#### Refinement

*R*[*F* ^2^ > 2σ(*F* ^2^)] = 0.040
*wR*(*F* ^2^) = 0.111
*S* = 1.03
2293 reflections
209 parameters
H-atom parameters constrained
Δρ_max_ = 0.34 e Å^−3^
Δρ_min_ = −0.32 e Å^−3^

### 

Data collection: *SMART* (Bruker, 2007 ▶); cell refinement: *SAINT-Plus* (Bruker, 2007 ▶); data reduction: *SAINT-Plus*; program(s) used to solve structure: *SHELXS97* (Sheldrick, 2008 ▶); program(s) used to refine structure: *SHELXL97* (Sheldrick, 2008 ▶); molecular graphics: *SHELXTL* (Sheldrick, 2008 ▶); software used to prepare material for publication: *SHELXTL*.

## Supplementary Material

Crystal structure: contains datablocks global, I. DOI: 10.1107/S1600536810031922/ng5003sup1.cif

Structure factors: contains datablocks I. DOI: 10.1107/S1600536810031922/ng5003Isup2.hkl

Additional supplementary materials:  crystallographic information; 3D view; checkCIF report

## Figures and Tables

**Table 1:** Selected bond lengths (Å)

Cu1—N1	2.016 (2)
Cu1—N3	2.016 (2)
Cu1—O1	2.678 (2)

**Table 2:** Hydrogen-bond geometry (Å, °)

*D*—H⋯*A*	*D*—H	H⋯*A*	*D*⋯*A*	*D*—H⋯*A*
N2—H2⋯O1^ii^	0.86	1.94	2.745 (4)	155
N4—H4⋯F1^iii^	0.86	2.26	2.874 (4)	128
N4—H4⋯O1^ii^	0.86	2.40	3.127 (4)	142

Symmetry codes: (ii) 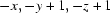; (iii) 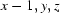.

**Fig. 1.**
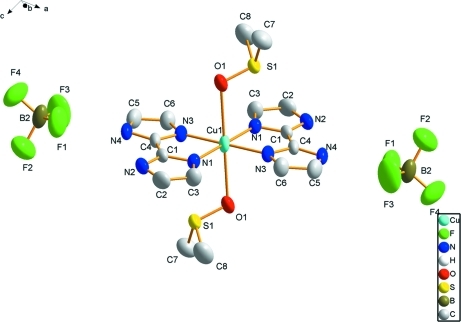
Perspective view of a basic unit of the title complex. Hydrogen atoms are omitted for clarity. Atoms are displayed as elliposoids at the 50% probability level.

**Fig. 2.**
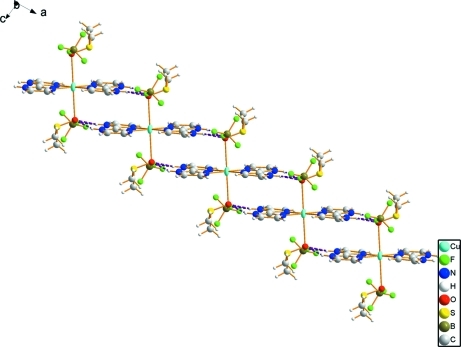
Step-like chain of [Cu(H2biim)2(DMSO)2]2+ (BF4)2 unit along b axis.

## supplementary crystallographic information

### Comment

Supramolecular complexes containing H_2_biim have been applied widely in molecular catalysis, photoelectric conversion materials and molecular recognition (Ding *et al.*, 2005) The ligand of H_2_biim has been widely studied and applied because of the diversity of their coordination and the strong ability to form hydrogen bonds as a multi-proton donor. The utilization of the coordination bonds of a transition metal ion, intermolecular hydrogen bonds and πi-πi packing interactions help to control the molecular arrangement (Burrows, 2004; Dai *et al.*, 2009). We focus on the synthesis of the biimidazole-metal complexes. Here we report a new complex [Cu(H_2_biim)_2_(DMSO)_2_](BF_4_)_2_ (1). Similar complexes {[Cu(H_2_biim)_2_(H_2_O)](SiF_6_)}.H_2_O (2), [Cu(H_2_biim)_2_](ClO_4_)_2_.2DMSO (Jin *et al.*,2010) and [Cd(H_2_biim)_3_](SiF_6_)(BF_4_)_2_.6EtOH (Gruia *et al.*,2007) will be compared here.

The title complex is composed of [Cu(H_2_biim)_2_(DMSO)_2_]^2+^ and two free BF_4_^-^ anions. Cu(II) atom is in the center of Jahn-Teller elongated octahedron. The equatorial positions are occupied by four nitrogen atoms of two bidentate H_2_biim molecules, while the axial positions are occupied by O atoms from two DMSO (Fig. 1). The [Cu(H_2_biim)_2_(DMSO)_2_]^2+^ unit stacks along the *b* axis to form a step-shaped infinite chain structure through πi-πi stacking and H-bonds(Fig.2).

The two identical distances Cu ··· O(DMSO) of 2.678 (2)Å are in the range of Cu—O coordination bond (from 2.522Å to 2.724 Å) (Tao *et al.*, 2002). The two identical Cu—N distances of 2.016 (2)Å are slightly shorter than those in [Cu(H_2_biim)_2_](ClO_4_)_2_.2DMSO [2.021 (2)Å and 2.018 (2) Å]. In the title complex there exist two types of hydrogen bonds, one is N—H···O formed between N—H group of the H_2_biim and oxygen atom of DMSO, the other is N—H···F formed between N—H group of H_2_biim and fluorine atom of BF_4_^-^. The DMSO molecule and BF_4_^-^ anion are located at both sides of the cation to form hydrogen bonds mentioned above. The face-to-face distance between the immidazole rings is 3.43Å with the dihedral angle of 3.718°, which suggests the existence of significant πi-πi interactions between them. There is a weak interaction Cu···S_DMSO_(3.458 Å) in complex (1).

The solvent plays an important role in the reaction of metal salt with 2,2'-bimidazole. Not only the configuration of the anions but also the coordination geometry of the cations are affected by the solvent. Complex 1 was prepared in the mixed solvent of ethanol and DMSO by the reaction of 2,2'- bimidazole with copper tetrafluoroborate with molar ratio 3:1. However, the ratio of ligand and metal in the cation [Cu(H_2_biim)_2_(DMSO)_2_]^2+^ of complex 1 is not consistent with the raw molar ratio, which may be related to the selectivity of solvent DMSO. Complex 2, {[Cu(H_2_biim)_2_(H_2_O)]SiF_6_}.H_2_O, was prepared by the similar method of preparing (1) except using solvent water (Jin *et al.*,2010). Due to the different solvents, both the cation and the anion in complex (1) and complex (2) are different. It is noted that in complex (1) the anion BF_4_^-^ was coming from starting material Cu(BF_4_)_2_ while in complex (2) the anion SiF_6_^2-^ was not, but was formed by the reaction of BF_4_^-^ with glass container in water. The complex [Cd(H_2_biim)_3_](SiF_6_)(BF_4_)_2_.6EtOH(Gruia *et al.*,2007) contains mixed cations SiF_6_^2-^and BF_4_^-^, which is related to the mixed solvent water and enthanol used in the reaction system.

The title complex is also similar to the following complexes: [Cu(H_2_biim)_2_](ClO_4_)_2_ (Aminou *et al.*,2004), [Cu(H_2_biim)_2_]Br_2_(Yang *et al.*,2008) and [Cu(H_2_biim)_2_](ClO_4_)_2_.2DMSO (Jin *et al.*,2010).

### Experimental

Cu(BF_4_)_2_.6H_2_O (0.1726 g, 1 mmol)dissolved in C_2_H_5_OH (5 ml) was added to a solution of H_2_biim (0.2010 g, 3 mmol) in C_2_H_5_OH (5 ml). The mixture was refluxed for 0.5 h, then 1 ml DMSO was added, stirring for another hour at room temperature, then filtered. Subsequent slow evaporation of the filtrate resulted in the formation of green crystals of the title complex after four weeks. Crystals suitable for single-crystal X-ray diffraction were selected directly from the sample as prepared. Analysis found(percentage): C 38.08, H 3.83, N 22.19; calculated:*C* 37.71, H 3.90, N 21.86.

### Refinement

Metal atom centers were located from the E-maps and other non-hydrogen atoms were located in successive difference Fourier syntheses. The final refinements were performed by full matrix least-squares methods with anisotropic thermal parameters for non-hydrogen atoms on F^2^.

The final refinements were performed with isotropic thermal parameters. All hydrogen atoms were located in the calculated sites and included in the final refinement in the riding model approximation with displacement parameters derived from the parent atoms to which they were bonded.

### Crystal data

**Table:**

[Cu(C_6_H_6_N_4_)_2_(C_2_H_6_OS)_2_](BF_4_)_2_	*Z* = 1
*M**_r_* = 661.71	*F*(000) = 335
Triclinic, *P*1	*D*_x_ = 1.643 Mg m^−^^3^
Hall symbol: -P 1	Mo *K*α radiation, λ = 0.71073 Å
*a* = 7.059 (1) Å	Cell parameters from 2039 reflections
*b* = 10.0721 (13) Å	θ = 2.2–27.1°
*c* = 10.3669 (15) Å	µ = 1.06 mm^−^^1^
α = 113.436 (2)°	*T* = 298 K
β = 96.860 (1)°	Block, green
γ = 92.000 (1)°	0.36 × 0.32 × 0.20 mm
*V* = 668.68 (16) Å^3^	

### Data collection

**Table:**

Bruker SMART CCD area-detector diffractometer	2293 independent reflections
Radiation source: fine-focus sealed tube	1885 reflections with *I* > 2σ(*I*)
graphite	*R*_int_ = 0.023
phi and ω scans	θ_max_ = 25.0°, θ_min_ = 2.2°
Absorption correction: multi-scan *SADABS*	*h* = −8→8
*T*_min_ = 0.701, *T*_max_ = 0.816	*k* = −11→11
3418 measured reflections	*l* = −12→11

### Refinement

**Table:**

Refinement on *F*^2^	Secondary atom site location: difference Fourier map
Least-squares matrix: full	Hydrogen site location: inferred from neighbouring sites
*R*[*F*^2^ > 2σ(*F*^2^)] = 0.040	H-atom parameters constrained
*wR*(*F*^2^) = 0.111	*w* = 1/[σ^2^(*F*_o_^2^) + (0.0545*P*)^2^ + 0.5099*P*] where *P* = (*F*_o_^2^ + 2*F*_c_^2^)/3
*S* = 1.03	(Δ/σ)_max_ = 0.001
2293 reflections	Δρ_max_ = 0.34 e Å^−^^3^
209 parameters	Δρ_min_ = −0.32 e Å^−^^3^
0 restraints	Extinction correction: *SHELXL97* (Sheldrick, 2008), Fc^*^=kFc[1+0.001xFc^2^λ^3^/sin(2θ)]^-1/4^
Primary atom site location: structure-invariant direct methods	Extinction coefficient: 0.048 (5)

### Special details

**Table:**

Geometry. All e.s.d.'s (except the e.s.d. in the dihedral angle between two l.s. planes) are estimated using the full covariance matrix. The cell e.s.d.'s are taken into account individually in the estimation of e.s.d.'s in distances, angles and torsion angles; correlations between e.s.d.'s in cell parameters are only used when they are defined by crystal symmetry. An approximate (isotropic) treatment of cell e.s.d.'s is used for estimating e.s.d.'s involving l.s. planes.
Refinement. Refinement of *F*^2^ against ALL reflections. The weighted *R*-factor *wR* and goodness of fit *S* are based on *F*^2^, conventional *R*-factors *R* are based on *F*, with *F* set to zero for negative *F*^2^. The threshold expression of *F*^2^ > σ(*F*^2^) is used only for calculating *R*-factors(gt) *etc*. and is not relevant to the choice of reflections for refinement. *R*-factors based on *F*^2^ are statistically about twice as large as those based on *F*, and *R*- factors based on ALL data will be even larger.

### Fractional atomic coordinates and isotropic or equivalent isotropic displacement parameters (Å^2^)

**Table:**

	*x*	*y*	*z*	*U*_iso_*/*U*_eq_	Occ. (<1)
Cu1	0.5000	0.5000	0.5000	0.0390 (2)	
F1	0.7595 (5)	0.2780 (3)	0.7673 (4)	0.1074 (11)	
F2	0.7494 (11)	0.2364 (7)	0.9569 (6)	0.133 (3)	0.671 (10)
F3	0.8593 (16)	0.0735 (12)	0.7739 (12)	0.131 (5)	0.671 (10)
F4	0.5500 (11)	0.1020 (9)	0.7667 (9)	0.111 (3)	0.671 (10)
F2'	0.676 (3)	0.0585 (13)	0.6485 (15)	0.153 (7)	0.329 (10)
F3'	0.577 (2)	0.1641 (18)	0.8495 (19)	0.114 (7)	0.329 (10)
F4'	0.873 (3)	0.121 (3)	0.844 (3)	0.137 (10)	0.329 (10)
N1	0.3310 (3)	0.6573 (3)	0.5939 (3)	0.0400 (6)	
N2	0.0728 (4)	0.7101 (3)	0.6960 (3)	0.0487 (7)	
H2	−0.0297	0.7002	0.7297	0.058*	
N3	0.3261 (3)	0.3835 (3)	0.5650 (3)	0.0392 (6)	
N4	0.0623 (4)	0.3776 (3)	0.6562 (3)	0.0472 (7)	
H4	−0.0392	0.4047	0.6943	0.057*	
O1	0.2552 (3)	0.4077 (3)	0.2573 (2)	0.0501 (6)	
S1	0.37073 (11)	0.34825 (10)	0.13515 (8)	0.0454 (3)	
B2	0.7268 (7)	0.1654 (5)	0.8039 (6)	0.0657 (13)	
C1	0.1883 (4)	0.6060 (4)	0.6387 (3)	0.0378 (7)	
C2	0.1466 (5)	0.8336 (4)	0.6911 (4)	0.0585 (10)	
H2A	0.0972	0.9235	0.7252	0.070*	
C3	0.3060 (5)	0.8018 (4)	0.6273 (4)	0.0526 (9)	
H3	0.3849	0.8663	0.6093	0.063*	
C4	0.1839 (4)	0.4588 (4)	0.6222 (3)	0.0377 (7)	
C5	0.1283 (5)	0.2444 (5)	0.6198 (4)	0.0579 (10)	
H5	0.0720	0.1651	0.6303	0.069*	
C6	0.2919 (5)	0.2493 (4)	0.5652 (4)	0.0516 (9)	
H6	0.3691	0.1732	0.5329	0.062*	
C7	0.2567 (7)	0.3869 (5)	−0.0058 (4)	0.0692 (11)	
H7A	0.1224	0.3562	−0.0226	0.104*	
H7B	0.3121	0.3360	−0.0902	0.104*	
H7C	0.2735	0.4895	0.0189	0.104*	
C8	0.3115 (7)	0.1570 (5)	0.0606 (5)	0.0762 (13)	
H8B	0.3505	0.1193	0.1304	0.114*	
H8C	0.3765	0.1122	−0.0202	0.114*	
H8A	0.1756	0.1363	0.0314	0.114*	

### Atomic displacement parameters (Å^2^)

**Table:**

	*U*^11^	*U*^22^	*U*^33^	*U*^12^	*U*^13^	*U*^23^
Cu1	0.0288 (3)	0.0399 (4)	0.0538 (4)	0.0093 (2)	0.0220 (2)	0.0197 (3)
F1	0.110 (2)	0.0760 (19)	0.165 (3)	0.0132 (16)	0.069 (2)	0.066 (2)
F2	0.163 (6)	0.137 (5)	0.080 (4)	−0.018 (4)	0.039 (4)	0.020 (3)
F3	0.130 (10)	0.099 (7)	0.197 (12)	0.083 (7)	0.096 (10)	0.067 (7)
F4	0.084 (4)	0.102 (6)	0.142 (7)	−0.037 (4)	−0.005 (5)	0.053 (5)
F2'	0.180 (15)	0.096 (9)	0.129 (11)	0.004 (8)	0.030 (10)	−0.013 (7)
F3'	0.095 (13)	0.124 (13)	0.143 (16)	0.020 (10)	0.085 (13)	0.056 (11)
F4'	0.098 (13)	0.120 (17)	0.18 (2)	0.012 (11)	−0.044 (14)	0.060 (15)
N1	0.0305 (13)	0.0407 (15)	0.0455 (15)	0.0044 (11)	0.0131 (11)	0.0119 (12)
N2	0.0365 (14)	0.0599 (19)	0.0431 (16)	0.0132 (13)	0.0190 (12)	0.0095 (14)
N3	0.0332 (13)	0.0446 (15)	0.0454 (15)	0.0063 (11)	0.0135 (11)	0.0217 (12)
N4	0.0333 (14)	0.066 (2)	0.0450 (16)	−0.0008 (13)	0.0155 (12)	0.0237 (14)
O1	0.0525 (14)	0.0504 (14)	0.0450 (13)	0.0066 (11)	0.0263 (11)	0.0113 (11)
S1	0.0382 (5)	0.0576 (6)	0.0371 (5)	0.0021 (4)	0.0142 (3)	0.0134 (4)
B2	0.051 (3)	0.052 (3)	0.105 (4)	0.009 (2)	0.034 (3)	0.037 (3)
C1	0.0261 (14)	0.0506 (19)	0.0314 (15)	0.0073 (13)	0.0100 (12)	0.0092 (14)
C2	0.056 (2)	0.048 (2)	0.059 (2)	0.0190 (18)	0.0190 (18)	0.0050 (18)
C3	0.050 (2)	0.042 (2)	0.064 (2)	0.0095 (15)	0.0198 (17)	0.0154 (17)
C4	0.0261 (14)	0.056 (2)	0.0328 (16)	0.0029 (13)	0.0103 (12)	0.0181 (14)
C5	0.055 (2)	0.065 (3)	0.065 (2)	−0.0041 (19)	0.0159 (18)	0.037 (2)
C6	0.050 (2)	0.051 (2)	0.063 (2)	0.0090 (16)	0.0183 (17)	0.0298 (18)
C7	0.089 (3)	0.070 (3)	0.053 (2)	0.015 (2)	0.015 (2)	0.026 (2)
C8	0.100 (3)	0.054 (3)	0.075 (3)	0.020 (2)	0.037 (3)	0.018 (2)

### Geometric parameters (Å, °)

**Table:**

Cu1—N1	2.016 (2)	N4—H4	0.8600
Cu1—N1^i^	2.016 (2)	N4—C4	1.335 (4)
Cu1—N3^i^	2.016 (2)	N4—C5	1.357 (5)
Cu1—N3	2.016 (2)	O1—S1	1.519 (2)
Cu1—O1	2.678 (2)	S1—C7	1.769 (4)
F1—B2	1.351 (5)	S1—C8	1.779 (4)
F2—B2	1.443 (8)	C1—C4	1.422 (5)
F3—B2	1.316 (9)	C2—H2A	0.9300
F4—B2	1.322 (8)	C2—C3	1.356 (5)
F2'—B2	1.529 (14)	C3—H3	0.9300
F3'—B2	1.213 (13)	C5—H5	0.9300
F4'—B2	1.23 (2)	C5—C6	1.353 (5)
N1—C1	1.328 (4)	C6—H6	0.9300
N1—C3	1.378 (4)	C7—H7A	0.9600
N2—H2	0.8600	C7—H7B	0.9600
N2—C1	1.340 (4)	C7—H7C	0.9600
N2—C2	1.353 (5)	C8—H8B	0.9600
N3—C4	1.331 (4)	C8—H8C	0.9600
N3—C6	1.365 (4)	C8—H8A	0.9600
			
F1—B2—F2	102.1 (5)	N3—C6—H6	125.3
F1—B2—F2'	92.2 (7)	N4—C4—C1	132.0 (3)
F2—B2—F2'	165.0 (7)	N4—C5—H5	126.6
F3—B2—F1	113.0 (7)	O1—S1—C7	107.35 (18)
F3—B2—F2	106.0 (7)	O1—S1—C8	104.93 (18)
F3—B2—F4	113.7 (8)	S1—O1—Cu1	107.75 (12)
F3—B2—F2'	71.8 (8)	S1—C7—H7A	109.5
F4—B2—F1	115.9 (6)	S1—C7—H7B	109.5
F4—B2—F2	104.4 (6)	S1—C7—H7C	109.5
F4—B2—F2'	64.6 (8)	S1—C8—H8B	109.5
F3'—B2—F1	114.3 (9)	S1—C8—H8C	109.5
F3'—B2—F2	67.7 (10)	S1—C8—H8A	109.5
F3'—B2—F3	132.6 (11)	C1—N1—Cu1	111.0 (2)
F3'—B2—F4	37.9 (7)	C1—N1—C3	106.2 (3)
F3'—B2—F2'	102.4 (11)	C1—N2—H2	126.1
F3'—B2—F4'	123.6 (18)	C1—N2—C2	107.7 (3)
F4'—B2—F1	114.2 (15)	C2—N2—H2	126.1
F4'—B2—F2	75.5 (12)	C2—C3—N1	108.3 (3)
F4'—B2—F3	31.1 (11)	C2—C3—H3	125.9
F4'—B2—F4	128.6 (14)	C3—N1—Cu1	142.8 (2)
F4'—B2—F2'	102.9 (11)	C3—C2—H2A	126.3
N1—Cu1—N1^i^	180.00 (16)	C4—N3—Cu1	111.3 (2)
N1^i^—Cu1—N3^i^	82.24 (10)	C4—N3—C6	105.6 (3)
N1^i^—Cu1—N3	97.76 (10)	C4—N4—H4	126.3
N1—Cu1—N3^i^	97.76 (10)	C4—N4—C5	107.5 (3)
N1—Cu1—N3	82.24 (10)	C5—N4—H4	126.3
N1—Cu1—O1	90.17 (9)	C5—C6—N3	109.4 (3)
N1^i^—Cu1—O1	89.83 (9)	C5—C6—H6	125.3
N1—C1—N2	110.5 (3)	C6—N3—Cu1	143.0 (2)
N1—C1—C4	118.0 (3)	C6—C5—N4	106.7 (3)
N1—C3—H3	125.9	C6—C5—H5	126.6
N2—C1—C4	131.6 (3)	C7—S1—C8	98.8 (2)
N2—C2—H2A	126.3	H7A—C7—H7B	109.5
N2—C2—C3	107.3 (3)	H7A—C7—H7C	109.5
N3^i^—Cu1—N3	180.0	H7B—C7—H7C	109.5
N3—Cu1—O1	87.32 (9)	H8B—C8—H8C	109.5
N3^i^—Cu1—O1	92.68 (9)	H8B—C8—H8A	109.5
N3—C4—N4	110.9 (3)	H8C—C8—H8A	109.5
N3—C4—C1	117.1 (3)		
			
Cu1—N1—C1—N2	−177.6 (2)	N3—Cu1—N1—C3	176.9 (4)
Cu1—N1—C1—C4	3.6 (3)	N3^i^—Cu1—N1—C3	−3.1 (4)
Cu1—N1—C3—C2	177.8 (3)	N3^i^—Cu1—N3—C4	−68 (100)
Cu1—N3—C4—N4	175.87 (19)	N3^i^—Cu1—N3—C6	107 (100)
Cu1—N3—C4—C1	−5.3 (3)	N3—Cu1—O1—S1	−129.93 (14)
Cu1—N3—C6—C5	−173.6 (3)	N3^i^—Cu1—O1—S1	50.07 (14)
Cu1—O1—S1—C7	−146.23 (18)	N4—C5—C6—N3	−1.1 (4)
Cu1—O1—S1—C8	109.39 (19)	O1—Cu1—N1—C1	82.3 (2)
N1^i^—Cu1—N1—C1	−142 (100)	O1—Cu1—N1—C3	−95.8 (4)
N1^i^—Cu1—N1—C3	40 (100)	O1—Cu1—N3—C4	−85.0 (2)
N1^i^—Cu1—N3—C4	−174.5 (2)	O1—Cu1—N3—C6	89.6 (4)
N1—Cu1—N3—C4	5.5 (2)	C1—N1—C3—C2	−0.4 (4)
N1^i^—Cu1—N3—C6	0.1 (4)	C1—N2—C2—C3	1.3 (4)
N1—Cu1—N3—C6	−179.9 (4)	C2—N2—C1—N1	−1.6 (4)
N1—Cu1—O1—S1	147.85 (14)	C2—N2—C1—C4	177.0 (3)
N1^i^—Cu1—O1—S1	−32.15 (14)	C3—N1—C1—N2	1.2 (4)
N1—C1—C4—N3	1.2 (4)	C3—N1—C1—C4	−177.6 (3)
N1—C1—C4—N4	179.7 (3)	C4—N3—C6—C5	1.2 (4)
N2—C1—C4—N3	−177.3 (3)	C4—N4—C5—C6	0.7 (4)
N2—C1—C4—N4	1.3 (6)	C5—N4—C4—N3	0.0 (4)
N2—C2—C3—N1	−0.5 (4)	C5—N4—C4—C1	−178.6 (3)
N3^i^—Cu1—N1—C1	175.1 (2)	C6—N3—C4—N4	−0.7 (4)
N3—Cu1—N1—C1	−4.9 (2)	C6—N3—C4—C1	178.1 (3)

Symmetry codes: (i) −*x*+1, −*y*+1, −*z*+1.

### Hydrogen-bond geometry (Å, °)

**Table:**

*D*—H···*A*	*D*—H	H···*A*	*D*···*A*	*D*—H···*A*
N2—H2···O1^ii^	0.86	1.94	2.745 (4)	155.
N4—H4···F1^iii^	0.86	2.26	2.874 (4)	128.
N4—H4···O1^ii^	0.86	2.40	3.127 (4)	142.

Symmetry codes: (ii) −*x*, −*y*+1, −*z*+1; (iii) *x*−1, *y*, *z*.
